# Voluntary Participation Mediates the Relationship Between Multi-Membership in Online Communities and Life Satisfaction Among Chinese Populations: A Gendered Perspective

**DOI:** 10.3390/bs14110976

**Published:** 2024-10-22

**Authors:** Xiaorui Huang, Mingqi Fu

**Affiliations:** 1 School of International Relations, Huaqiao University, Xiamen 361021, China; huangxiaorui@hqu.edu.cn; 2 School of Public Administration, Central South University, Changsha 410012, China

**Keywords:** digital mental health, gender, life satisfaction, online engagement, voluntary participation

## Abstract

Whether and how multi-membership in online communities might relate to life satisfaction within the Chinese population remain unclear. This study adopts a gendered perspective to explore the mediating role of voluntary participation in the relationship mentioned above based on a cross-sectional analysis of 2558 respondents from the 2019 Chinese Social Survey (CSS). Multivariable regressions and a mediation analysis were adopted for analyses. The findings reveal that a higher level of multi-membership in online communities is associated with greater life satisfaction for both males (B = 0.31, SE = 0.11) and females (B = 0.10, SE = 0.02). Specifically, the positive relationship is partially mediated (6.6%) by increased voluntary participation among females, where involvement in multiple types of online communities correlates with a heightened likelihood of engaging in voluntary activities (B = 0.006, Z = 3.910), which in turn contributes to higher levels of life satisfaction (B = 0.114, Z = 2.760). However, voluntary participation does not exhibit a significant mediating role in the relationship between multi-membership and life satisfaction among males. These findings provide valuable insights into the intricate ways in which online interactions can affect voluntary participation and life satisfaction, underscoring the importance of considering gender differences in these dynamics.

## 1. Introduction

The rapid advancement of information and communication technologies (ICTs) has profoundly impacted various aspects of individual life [1]. In particular, the internet could serve as an integrated medium that facilitates long-distance communication through text, images, and audio [2], thereby to some extent changing social interaction patterns. Previous studies have demonstrated that online interactions, mainly within online communities [3], substantially enrich the social networks of individuals and enhance information exchange and cooperation [4]. These online communities often replicate physical social networks and are established around specific relationships, including kinship, friendship, professional ties, shared interests, leisure activities, neighborhood connections, and the protection of rights [5]. Compared with in-person communities, online communities tend to have a more dynamic and transient structure [6,7], characterized by loose boundaries that allow members to enter or exit with greater flexibility. Recently, a growing number of individuals have sought connections across diverse interests and support networks, leading to membership in multiple communities, sometimes exceeding 10 [8]. This multi-membership has emerged as a predominant feature of modern netzines, potentially exerting multidimensional impacts on individuals’ daily life, such as on their psychological well-being. While previous studies have substantially examined the effects of increased online communication on individuals’ psychological responses [9,10], few have considered the implications of multi-membership in different types of online communities, despite the importance of these findings for developing psychological well-being programs in a more prosperous digital era.

## 2. Literature Review

### 2.1. Relationship Between Engagement in Online Communities and Life Satisfaction

Life satisfaction is defined as individuals’ cognitive evaluation of the overall quality of their lives and is considered a central driver of emotional, social, and behavioral outcomes [11,12]. Previous studies focusing on the frequency of individuals’ engagement with online communities have yielded mixed results regarding its relationship with life satisfaction. While prior research suggests that more frequent engagement in online communities is positively correlated with greater life satisfaction [13]—by facilitating remote communication, extending interpersonal interactions, enlarging social support network, and maintaining dispersed friendships—other studies argue that deeper engagement in online activities may foster feelings of overwhelm or burnout, especially if it replaces face-to-face interactions, thereby reducing life satisfaction [14,15]. Serving as a crucial indicator of individuals’ social interaction patterns, multi-membership in online communities might also significantly influence psychological responses such as life satisfaction [11]. Unlike engagement frequency, which focuses on the depth and consistency of involvement in a specific community, multi-membership highlights the breadth of connections across various communities [7,9]. In this context, the positive effects of diverse relationships and varied sources of support can contrast with the potential negative impacts of feelings of fragmentation, making the discussion more nuanced. However, empirical evidence on this topic is still lacking.

Meanwhile, in-person community evidence also reveals a complex relationship between multi-membership and life satisfaction. On the one hand, participation in a variety of communities could improve individuals’ access to multiple identity resources, potentially enhancing their self-image and improving life satisfaction [16,17,18]. Evidence from multiple traumatic contexts indicates that individuals with multiple group memberships exhibit greater resilience in the face of adversity, often leading to enhanced health and well-being after significant life changes [19]. One the other hand, increased engagement in diverse communities may correlate with a more negative perception of one’s life. For instance, multi-membership could expose individuals to more negative or aggressive interactions, which are detrimental to life satisfaction [20]. Furthermore, individuals assuming different roles in various communities might also experience cognitive dissonance or become overwhelmed by conflicting responsibilities, resulting in lower levels of life satisfaction [21]. The inconsistent findings could be attributed to differences in methodologies, including sample selection and study design, as well as the sociocultural contexts in which the communities are situated. Given that individuals might experience diverse online community engagements and more frequent membership changes, it is reasonable to hypothesize that the relationship between multi-membership in online communities and individual life satisfaction may be even more pronounced than that observed in in-person communities.

### 2.2. A Gender-Specific Mediating Role of Voluntary Participation

#### 2.2.1. A Synthesized Theoretical Model

In addition to direct psychological arousals, the relationship between multi-membership in online communities and life satisfaction may be mediated by behavioral changes, such as evolving patterns of voluntary participation. Volunteering is defined as the act of offering time and skills to help others or support a cause without expecting financial compensation; it typically involves community service, charitable work, or participation in nonprofit organizations [22]. According to the Citizenship Voluntary Model (CVM), volunteering is a social behavior influenced by three key variables: motivation, participation resources, and social mobilization [23]. In this context, multi-membership in online communities as a form of social interaction may alter individuals’ sense of responsibility and efficacy in volunteering, affecting their self-perception and information processing, as well as their resource networks, including monetary, time, and skill reserves. Also, engaging with diverse online communities may simultaneously correlate with opportunities for participation in volunteering activities. Although the CVM was originally developed to investigate voluntary participation in in-person contexts, numerous studies have demonstrated its validity in describing behaviors and determinants of voluntary participation in digital environments [24,25].

Once voluntary behaviors are initiated, individuals’ life satisfaction may subsequently change. According to the Hedonic and Eudaimonic Well-being framework (HEWM), well-being outcomes resulting from specific behaviors are a combination of hedonic well-being—derived from sensory pleasure, material gains, and emotional gratification—and eudaimonic well-being, which stems from living a meaningful life, purpose, and self-realization [26]. Voluntary participation can enhance hedonic well-being by providing immediate joy or positive feelings from helping others, as well as eudaimonic well-being by aligning with personal values and promoting growth. 

Therefore, this study synthesizes the CVM and HEWM to analyze the continuous process linking multi-membership in online communities, voluntary participation, and life satisfaction. It is noteworthy that this theoretical framework can be enriched by a gendered perspective. According to Gender Socialization Theory, men and women are often socialized differently, resulting in distinct norms, behaviors, and expectations [27]. Consequently, gender shapes how individuals process information about the world and maintain personal and societal dynamics [28]. In this context, gender may influence individuals’ motivation levels, their participation resources, and their engagement in social mobilization following online interactions, shaping distinct voluntary behaviors as an outcome. Furthermore, voluntary participation may resonate with men’s and women’s gender identities, maintaining a sense of consistency to varying extents and contributing to different levels of life satisfaction.

**Figure 1:**
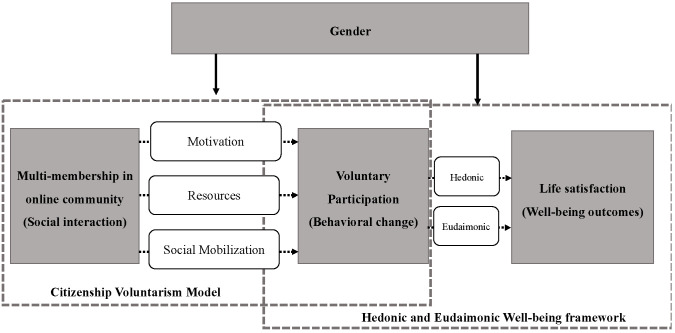
A gendered volunteering bridge model.

Thus, the current study constructs a gendered volunteering bridge model to explore and support the mediating role of voluntary participation in the relationship between multi-membership in online communities and life satisfaction (see Figure 1). Prior research by Wacker posits that an effective model should satisfy criteria including parsimony, fecundity, internal consistency, empirical riskiness, and abstraction [29]. Accordingly, the CVM treats voluntary behaviors as an outcome variable, while the HEWM considers individual behaviors as an independent variable. This gendered volunteering bridge model synthesizes these models in a sequential order to elucidate the continuous process from online community engagement to life satisfaction, thus demonstrating parsimony and good internal consistency. Additionally, the model incorporates a gendered perspective to enhance the fecundity of the synthesized framework, as each relationship has been examined and supported by empirical evidence. Ultimately, this gendered volunteering bridge model aims to provide an integrated explanatory framework for the complex interplay between online community engagement and life satisfaction outcomes, incorporating a novel gendered perspective and achieving the virtue of abstraction. Therefore, we believe this model offers a rational and insightful understanding of the relationship between online community engagement and life satisfaction.

#### 2.2.2. Empirical Evidence on the Relationship Between Online Interaction, Voluntary Participation, and Life Satisfaction

The CVM suggests that social interactions, including multi-membership in online communities, significantly impact individuals’ volunteering behaviors, but empirical evidence remains inconclusive regarding the direction of this relationship. On one hand, macro-level studies suggest that the emergence of Internet communication has created a flatter world, thereby expanding organizational opportunities for volunteering activities [30]. Recent years have witnessed a notable rise in online volunteering—examples include online tutoring, virtual fundraising, and skill-based support for nonprofits, while many in-person initiatives have transitioned to digital platforms [31]. In the United States, approximately 20–30% of residents engage in online volunteering or are motivated by digital opportunities [32]. At the micro-level, studies across various nations indicate a significant positive correlation between internet usage and voluntary participation [33,34]; for instance, a study involving the U.S., U.K., France, and Canada found that frequent users of online communication are three times more likely to engage in volunteer activities compared to those involved in non-interactive online activities [35]. It is possible that deeper engagement in online interactions may enhance their motivation for volunteering and reserve essential resources like communication skills [36,37]. Conversely, some studies argue that intensive Internet use may hinder volunteer participation due to time constraints, with Putnam noting a decline in community engagement as virtual activities proliferate [38]. Evidence from Switzerland also indicates that excessive Internet engagement may reduce volunteering likelihood [39], partly due to decision fatigue and decreased focus [40]. The inconsistent findings may be due to variations in how online interaction is defined and assessed, as limited attention has been given to the specific characteristics of multi-membership. This gap highlights the need for further research to examine the underexplored effects of multi-membership in online communities on volunteering. 

Regarding the relationship between volunteering and life satisfaction, empirical evidence is mixed, with researchers suggesting a positive, negative, or nonsignificant correlation. Many studies indicate that volunteering is a vital source of physical, social, and emotional well-being, contributing to psychological outcomes, including life satisfaction across life stages [41,42]. Particularly among older individuals, research from various regions—including Europe [43], Asia [44], and South America [45]—supports the notion that volunteering enhances life satisfaction through various channels, such as the enjoyment of helping others and increased self-appreciation. However, certain circumstances, such as crisis line volunteering, can negatively impact mental well-being due to high workloads and exposure to distressing information [46]. Often, crisis line volunteers report low personal accomplishment and poor life perceptions [47]. Meanwhile, prior research also indicates that irregular volunteering may adversely affect subjective happiness [48], while some studies find a nonsignificant relationship between volunteering and psychological well-being among older adults [49], attributing improved well-being more to role identity than volunteering itself. These inconsistencies highlight a need for further investigation into the nuanced interplay between volunteering and life satisfaction, particularly regarding the contextual and demographic factors that may influence these outcomes.

It is noteworthy that gender may moderate the relationships between online social interactions, voluntary participation, and life satisfaction. Previous research has explored gender differences in online communication and volunteering. For instance, a Swiss study found that men who frequently engage in online communication are more likely to volunteer than women, possibly due to men’s higher self-perceived Internet skills [31]. Another study supports this notion of male preference, assuming that men are more easily motivated by environmental information whereas women are generally more predisposed to helping others [50]. Yet, evidence from 27 countries indicates that this gender difference may be of marginal significance [34]. Conversely, other studies suggest women might be more motivated by online interactions, as they exhibit skepticism towards online information, which can be mitigated through diverse communication [51]. Despite these gender differences, prior studies generally agree that female volunteers experience greater reductions in depressive symptoms compared to males [52]. Evidence for volunteering at church resulting in a greater sense of well-being was found only among women, but not among men [53]. Therefore, it is reasonable to say that men and women might differ in terms of voluntary participation’s mediating role in the relationship aforementioned, but with its preference being debated. 

In summary, existing research highlights the complex interplay among online interaction, voluntary participation, and life satisfaction, suggesting a moderation mechanism in which gender influences these relationships. However, the nuances of these interactions remain unclear, particularly with regard to the unique social interaction pattern of multi-membership in online communities, indicating a need for further exploration to inform policies aimed at enhancing life satisfaction in the digital age.

## 3. Aims and Hypothesis

Understanding the relationship between online community engagement and life satisfaction, along with its underlying mechanisms, is particularly salient in China. In recent years, Chinese populations have shown a growing trend toward increased life satisfaction [54], coinciding with a rapid rise in Internet users. According to data from the National Bureau of Statistics of China, the number of Internet users has doubled to 989 million as of 2020, with a usage rate of instant messaging apps like WeChat and QQ at 90.7% [55]. This growth has led to the establishment of diverse online communities, which may uniquely influence individuals’ daily lives and psychological outcomes, including life satisfaction. During this process, volunteering has been experiencing significant growth, but the proportion of regular voluntary participation is still relatively low compared to Western countries, together with significant gender distinctions [56]. Therefore, this study aims to investigate the relationship between multi-membership in online communities and life satisfaction among the Chinese population, focusing on the mediating role of voluntary participation and potential gender differences. 

Our first hypothesis addresses the relationship between multi-membership in online communities and individual life satisfaction. We hypothesize two possible outcomes. Building on prior evidence suggesting that more frequent online interaction fosters interpersonal connections [13] and multi-membership in communities enhances resource acquisition and self-images [16,17,18,19],

**H1a.**  *suggests that engagement in a greater number of different types of online communities is associated with higher levels of life satisfaction.*

In contrast, due to the potential risks of burnout and negative information exposure resulting from intensive online interaction and multi-membership [14,15,20,21],

**H1b.**  *Posits that engagement in a greater number of different types of online communities is associated with lower levels of life satisfaction.*

To disentangle these hypotheses, we will examine the impact of multi-membership on life satisfaction using multi-variable regression models. If the results show a positive and significant relationship between multi-membership and life satisfaction (e.g., positive regression coefficients), this will support H1a; otherwise, it will support H1b.

Our second hypothesis pertains to the mediating role of voluntary participation in the relationship. Drawing on the synthesized volunteering bridge model (See Figure 1), we hypothesize that

**H2.**  *Voluntary participation partially mediates the relationship between multi-membership in online communities and life satisfaction.*

More specifically, considering possible growth in voluntary motivation and resource availability following more intensive online interaction [33,34,35,36,37],

**H2a.**  *Posits that greater multi-membership in online communities enhances the likelihood of voluntary participation, which in turn contributes to improved life satisfaction;*

Alternatively, informed by studies on decision fatigue and time constraints [38,39,40],

**H2b.**  *Argues that greater multi-membership in online communities reduce the likelihood of voluntary participation, leading to lower levels of life satisfaction. *

To test these alternative mediation hypotheses, we will conduct a mediation analysis, first testing whether multi-membership predicts higher or lower voluntary participation. If a significant positive association is found, H2a. would be supported. If a negative association is found, it would support H2b. If the indirect effect of multi-membership on life satisfaction through voluntary participation is non-significant, H2 would be denied. 

Finally, we incorporate a gendered perspective into the mediation model. According to Gender Socialization Theory [27], men and women differ in their social norms, behaviors, and expectations, as well as in how they process information and maintain personal and societal relationships. Previous research has also identified gender differences in volunteering behavior following online interaction [31,51]. Thus, we hypothesize that

**H3.**  *The significance and/or magnitude of the mediating effect of voluntary participation on the relationship between multi-membership in online communities and life satisfaction differs between men and women. *

To test H3., we will employ a multi-group mediation analysis to estimate the mediation model separately for males and females and compare the mediation effects across these two groups. If we find that the mediation effect is significant for both genders but the size of the effect is larger for one gender, or the mediation is significant only for one gender, H3. would be supported. If there is no significant difference between genders in the mediation effect, this would refute H3. 

## 4. Methods

### 4.1. Data Sources and Respondents

Data in this study were obtained from the 2019 Chinese Social Sciences Comprehensive Survey (CSS) conducted by the institute of Sociology at the Chinese Academy of Social Science. The CSS is a nationwide large-scale longitudinal survey initiated in 2005 and followed up every two years. It employs multistage stratified probability sampling strategies to recruit respondents from 151 cities and 31 provinces across China. Detailed information regarding ethical approval, sampling design, informed consent, response rate, and survey content could be found elsewhere [57]. The CSS collects a wide range of information, encompassing individuals’ multifaceted living circumstances, including social values, leisure activities, and physical and mental well-being [56]. In particular, the 2019 wave of the survey was the first time respondents were asked about their participation in online communities over the last two years. Online communities were primarily identified through groups on three major social media platforms, WeChat, Weibo, and QQ, which dominate the Chinese social media landscape and offer a range of functionalities for creating different types of online communities [55]. The 2019 wave of the CSS recruited over 10,000 respondents. For the purposes of this study, individuals with missing information on online community engagement (N = 143), life satisfaction (N = 1) and voluntary participation (N = 7581) were excluded. As a result, a total of 2558 individuals were included in the analysis. 

### 4.2. Measures

**The multi-membership in online communities** in this study was assessed with the questions “Have you joined each of the following online community in the last two years”. Respondents indicated their participation with a score of 1 for “yes” and 0 for “No”. A total of 12 community types were listed, including relatives, friends, neighbors, colleagues, religions, fellow villagers, alumni, interest groups, public-welfare associations, industries/associations, right protection, and other categories. The scores of these twelve questions were summed to indicate the level of individual engaged in different types of online communities. The total score ranged from 0 to 12, with a higher score representing a greater level of multi-membership.

**Voluntary participation** in this study was treated as a binary variable, indicating individuals’ voluntary experiences in the three months prior to the interview. Respondents were asked “How many times you have participated in voluntary activities in the past three months”. Responses of one and more were coded as 1 (Yes), while those indicating no participation were coded as 0 (Never).

**Life satisfaction** was measured by the extent to which individuals perceive their life as satisfactory. Respondents were asked with the question, “Generally speaking, to what extent you feel satisfied with your life?” with a 10-point scale. A higher score represents greater life satisfaction. 

**Gender differences** were categorized as male and female in the context of the relationship between online community engagement, voluntary participation, and life satisfaction. 

**Other covariates** included education level (less than lower secondary/upper secondary or vocational/tertiary), residence (urban/rural), marital status (single/married), subjective socioeconomic status (lower than ordinary level/ ordinary level/ higher than ordinary level), political status (communist/ non-community party member/none), and age (continuous variable).

### 4.3. Statistical Analyses

Descriptive analyses were conducted for all variables within the total sample, as well as separately for the female and male samples. For categorical variables, we reported the number and proportion of respondents, while for continuous variables, we provided the mean value and standard deviation. To determine the significance of differences in sample distribution between males and females, chi-square tests were performed for categorical variables and *t*-tests for continuous variables.

Next, we used a multivariate regression model to examine the relationship between the multi-membership in online communities and voluntary participation with life satisfaction. This analysis controlled for various covariates, including education, residence, marital status, subjective SES, age, and political status. Regressions were conducted for the total sample, as well as separately for the female and male samples. Standardized coefficients and the standard error were reported. 

Third, to examine the mediating role of voluntary participation in the relationship between the multi-membership in online communities and life satisfaction in both female and male samples, we utilized bootstrapping methods to obtain point estimates of the coefficients. Sobel–Goodman Mediation tests and bootstrap mediation tests were performed using the sgmediation command. This command assesses mediation effects according to Baron and Kenny’s methods [58], which require the following conditions to be met: (1) the independent variable significantly affects the mediator; (2) the independent variable significantly affects the dependent variable in the absence of the mediator; (3) the mediator has a significant unique effect on the dependent variable; and (4) the effect of the independent variable on the dependent variable is significantly reduced upon the addition of the mediator to the model. All analyses were conducted in Stata version 14.0.

All methods were carried out in accordance with relevant guidelines and regulations. Ethical review and approval were waived for this study, as we used the open database of the Chinese Social Survey (CSS) and did not include experimentation that may bring possible risk of harm to participants.

## 5. Results

Table 1 presents the key characteristics of the sample studied. Out of the 2558 respondents, 51.21% were male, and 58.52% (22.44% + 36.08%) had attained at least an upper secondary education. The average age of the respondents was 41.44 years old (SD = 14.53), with a majority residing in urban areas (63.1%) and being married (71.19%) at the time of the interview. Notably, about half of the respondents (45.82%) perceived their socioeconomic status as below average. Regarding online activities, respondents were involved in an average of 3.77 online communities (SD = 2.56), and over half of them (50.35%) had participated in voluntary activities. Overall, the respondents reported a high level of life satisfaction (mean = 7.43, SD = 2.03).

Bivariate correlation analyses indicated significant gender disparities in educational attainment, marital status, political affiliation, and age among respondents. Notably, the proportion of females with higher education (n = 289, 23.16%) and identifying as communist (377, 30.21%) was notably lower than that of males. Conversely, a greater proportion of females were married (965, 77.32%), though they were generally younger than their male counterparts (mean age =40.48, SD = 14.17). No significant differences were observed in terms of the level of multi-membership in online communities, voluntary participation, or life satisfaction. Detailed information could be found in Table 1.

Table 2 delves into the correlation between multi-membership in online communities, voluntary participation, and life satisfaction. Among female participants, those engaged in more types of online communities reported higher levels of life satisfaction (B = 0.10, SE = 0.02). In addition, increased participation in voluntary activities was positively linked to life satisfaction (B = 0.31, SE = 0.11). Conversely, for male respondents, voluntary participation did not show a significant association with life satisfaction (B = 0.21, SE = 0.11), although a broader range of online community engagement was connected to higher levels of life satisfaction (B = 0.06, SE = 0.02). 

As shown in Table 3, there is evidence of a mediating effect of voluntary participation in the relationship between multi-membership in online communities and life satisfaction among females. Specifically, involvement in a wider variety of online communities was associated with an increased likelihood of participating in voluntary activities (B = 0.006, Z = 3.910), which in turn contributed to higher levels of life satisfaction (B = 0.114, Z = 2.760). The Sobel–Goodman mediation test (refer to Table 4) further confirmed the significance of this mediation effect, indicating that the heightened voluntary participation explained 6.6% of the total effect of multi-membership in online community on enhanced life satisfaction. However, no significant mediation was observed among males. 

## 6. Discussion

This study adopted a gender-based approach to explore the relationship between individuals’ multi-membership in online communities and their life satisfaction while also examining the potential mediating role of voluntary participation. Our findings revealed that a greater level of multi-membership in online communities was associated with enhanced life satisfaction for both females and males. However, the significant mediating role of voluntary participation was only found among females, as a higher level of multi-membership in online communities was associated with increased likelihood of volunteering only among females. These results provide valuable insights into the nuanced impact of social media and online interactions on life satisfaction, emphasizing the importance of considering gender differences in these dynamics. Several findings of this study warrant further discussion. 

First, this study found that engagement in more different types of the online communities is associated with higher levels of life satisfaction. Previous studies have noted that extensive online social networking can enhance social support and relationships, thereby improving mental health outcomes [59,60]. Our study further validates the influence of multi-membership in more online communities, with possible explanations on two channels. From a motivation perspective, being part of multiple communities can bolster one’s social identity, allowing individuals to express various facets of their personality and interests [61]. A multifaceted social identity can enhance feelings of self-worth and belonging. Additionally, interacting with people from diverse backgrounds and with different viewpoints can broaden perspective, promote empathy, and enhance personal growth, all of which are linked with improved life satisfaction [62]. From a social mobilization and resource reservation perspective [11], engaging in more types of online communities may provide access to a broader network of support, as well as additional resources and information, contributing to a more stable and positive sense of well-being. Thus, it is reasonable for this study to suggest that online communities play an increasingly vital role in individuals’ social networking. Fostering a richer, more supportive, and more stimulating online environment might be crucial for enhancing life satisfaction.

Second, we found that increased voluntary participation mediates the positive relationship between online community engagement and life satisfaction among females. Based on our gendered volunteering bridge model, we found that engaging in more different types of online communities aligns with the social roles of females and encourages their participation in voluntary activities. When individuals engage in volunteering, women might form meaningful connections and relationships that enhance their sense of belonging, which subsequently boosts life satisfaction [52]. However, contrary to previous studies [31,50], we found that voluntary participation did not significantly impact men’s life satisfaction. This might be due to men deriving satisfaction from other sources, such as career achievements and physical activities, potentially undervaluing the impact of their volunteer efforts. Differences in volunteer motivations could also play a role. Men are more likely to engage in volunteering for social functional motivations, making it more challenging for men to enhance life satisfaction through volunteering without aligning it with their functional goals [63]. Thus, our study underscores the nuanced relationship between online community engagement, voluntary participation, and life satisfaction and urges that a gender-specific perspective is crucial for developing targeted strategies to enhance life satisfaction through Internet use and voluntary participation. 

Meanwhile, gender differences in the mediation effects may stem from the relationship between multi-membership in online communities and voluntary participation. In contrast with previous research positing a male preference in volunteering in a context of online engagement [31], this study found that positive effect is significantly only among females. Research indicates that women often use social networks to build relationships, while men tend to focus on task-oriented interactions [27]. Consequently, men’s engagement in online communities may not translate as directly into volunteerism. Additionally, from an information processing perspective, a higher level of multi-membership in online communities may allow females to cross-validate volunteering opportunities, thereby enhancing their likelihood of participation [51]. Furthermore, women may excel at utilizing their online networks for offline activities, including volunteering. The skills and connections cultivated within these online communities can enhance their ability to discover and engage in volunteer opportunities [64]. Notably, previous research indicates nearly half (43.82%) of female volunteers in China access information about volunteering opportunities through interpersonal communication via online media [65]. Therefore, this study suggests that the diversity and connectivity offered by multi-membership in online communities might enhance women’s ability to participate in volunteer activities and the distinct ways in which men and women engage with these online platforms highlight the necessity for tailored strategies that leverage women’s strengths in relationship building and information processing. 

### 6.1. Strengths and Limitations

This study is one of the first to examine the relationship between the multi-membership in online activities, voluntary participation, and life satisfaction from a gendered perspective. The findings indicate that a greater diversity of online communities significantly enhances overall life satisfaction. However, the positive relationship between multi-membership in online communities and the likelihood of voluntary participation, as well as the mediating effect of voluntary participation in this relationship, was found to be significant for females but not males. These findings reveal the nuanced and gender-specific ways in which social media and online interactions can affect life satisfaction, underscoring the need to consider gender differences in these dynamics.

However, several limitations should be acknowledged. Firstly, although the sequence of engaging in online communities and voluntary activities precedes life satisfaction as an outcome, the data were collected through a single cross-sectional survey, making it difficult to validate the longitudinal relationships between variables. Future studies employing longitudinal data are essential. Secondly, other covariates, such as personality traits, which might impact individuals’ life satisfaction, were not controlled for in this study. Thirdly, this study focused solely only on the number of types of online communities that individuals engage in without examining the distinct characteristics of different community clusters. For example, communities based on interpersonal relationships, such as those involving friends, family members, and relatives, might have different impacts on life satisfaction compared to communities established in a professional context. Additionally, we acknowledge that while we assume the diversity of engaged communities contributes to life satisfaction outcomes, the varying number of communities within each type may not have been fully considered, potentially introducing some estimation bias. Therefore, we believe that more detailed investigations into the differences and clusters among online communities are essential for future research. 

### 6.2. Theoretical Contribution and Practical Implications

By synthesizing insights from the Citizen Voluntary Model, the Hedonic and Eudaimonic Well-being framework, and Gender Socialization Theory, this study developed and validated a gendered volunteering bridge model. This model provides a more comprehensive understanding of how gender moderates the relationship between multi-membership in online communities, voluntary participation, and life satisfaction. It advances existing theories by incorporating gender-specific nuances in voluntary behavior and psychological outcomes, thereby addressing gaps in both gender studies and online interaction research. Perhaps, future studies incorporating qualitative or comparative evidence from different cultural contexts could be conducted to further validate this framework. Our findings extend the literature on the mechanisms through which online interactions influence well-being, supporting a more nuanced perspective that views volunteering not merely as an outcome but as a mediating process linking online community engagement and subjective well-being, particularly for females.

Moreover, the findings of this study have practical implications for promoting psychological well-being in the digital era. First, we suggest that encouraging voluntary participation within online communities could be an effective strategy for enhancing well-being. Mental health practitioners and community organizers could design programs that leverage multi-membership involvement as a platform to increase volunteering among online users, which could be vital for digital health support frameworks. Second, given that the mediation effect is significant only among females, programs aimed at improving life satisfaction could be tailored to address the specific psychological and behavioral needs of women. Facilitating access to diverse online groups and encouraging volunteering in these spaces may be especially beneficial for women’s psychological well-being. 

## 7. Conclusions

Using a sample of 2558 adults from China, this study is among the first to examine the mediating effect of increased voluntary participation on the relationship between multi-membership in online communities and life satisfaction from a gendered perspective. The findings indicate that multi-membership, an increasingly prevalent characteristic of online interactions, is positively associated with life satisfaction among Chinese netizens. Furthermore, increased opportunities for voluntary participation accounted for approximately 7% of this relationship, but the mediation effect was significant only for females, not males. This study proposes that the nuanced relationship between multi-membership in online communities, voluntary participation, and life satisfaction can be explained by the newly developed gendered volunteering bridge model. The findings underscore the importance of facilitating access to diverse online groups to promote mental health outcomes and encourage the development of tailored strategies for promoting volunteering, with a particular focus on prioritizing efforts for female netzines.

## Figures and Tables

**Table 1:** Descriptive data on socio-demographics, multi-membership in online communities, voluntary participation, and life satisfaction (N = 2558).

		Overall Sample (N = 2558)		Male (n = 1310)		Female (n = 1248)	
		N (Mean)	% (SD)	N (Mean)	% (SD)	N (Mean)	% (SD)
Education level *	Less than lower secondary	1058	41.36	382	30.13	679	54.33
	Upper secondary or vocational	574	22.44	294	22.44	280	22.44
	Tertiary	923	36.08	634	48.40	289	23.16
Residence	Urban	1614	63.1	781	59.62	833	66.75
	Rural	944	36.9	529	40.38	415	33.25
Marital Status *	Single	737	28.81	454	34.66	283	22.68
	Married	1821	71.19	856	65.34	965	77.32
Subjective SES *	Lower than ordinary level	1172	45.82	607	46.84	565	45.27
	Ordinary level	1104	43.16	561	43.29	543	43.51
	Higher than ordinary level	249	9.73	128	9.88	121	9.70
Politics Status *	Communist	871	34.05	494	37.71	377	30.21
	Non-communist party member	12	0.47	7	0.53	5	0.40
	None	1675	65.48	809	61.76	866	69.39
Multi-membership in Online Communities		3.77	2.56	3.75	2.58	3.79	2.55
Age *		41.44	14.53	42.37	14.82	40.48	14.17
Engaged in Voluntary Activities	Yes	1288	50.35	633	48.32	637	51.04
	No	1270	49.65	677	51.68	611	48.96
Life satisfaction		7.43	2.03	7.46	2.04	7.39	2.02

Notes: * means significant difference between sampled men and women in the corresponding item. For continuous variables, *t* tests were conducted, and for categorical variables, ANOVA analyses were conducted.

**Table 2:** Multivariate linear regression analysis of life satisfaction on the multi-membership in online community and voluntary participation (N = 2558).

	Total Sample		Male		Female	
	B	SE	B	SE	B	SE
Multi-membership in Online Communities	0.08 ***	0.02	0.06 **	0.02	0.10 ***	0.02
Engaged in Voluntary Activities (Ref: Uninvolved)	0.26 ***	0.08	0.21	0.11	0.31 **	0.11

Notes: For each of the variable, B refers the regression coefficient, SE refers the standard error. Covariates were controlled. *** *p* < 0.001; ** *p* < 0.01.

**Table 3:** The mediation effect of increased voluntary participation probability in the relationship between multi-membership in online communities and life satisfaction.

	Male			Female		
	B	SE	Z	B	SE	Z
Total effect	0.061	0.022	2.81 **	0.103	0.022	4.62 ***
Direct effect	0.059	0.022	2.72 **	0.096	0.022	4.30 ***
Indirect effect	0.002	0.002	1.32	0.007	0.003	2.260 *
X-M	0.010	0.005	1.85	0.022	0.006	3.910 ***
M-Y	0.211	0.112	1.88	0.315	0.114	2.760 **

Notes: X refers to independent variable (multi-membership in online community); M refers to mediator (voluntary participation); Y refers to outcome variable (Life satisfaction), and covariates were controlled and omitted. B refers the regression coefficient, SE refers the standard error, and Z refers the value of the Z test. *** *p* < 0.001; ** *p* < 0.01; * *p* < 0.05.

**Table 4:** Results of the Sobel–Goodman mediation tests among the female sample.

	B	SE	Z	*p* > |Z|
Sobel	0.007	0.003	2.254	0.024
Aroian	0.007	0.003	2.206	0.027
Goodman	0.007	0.003	2.305	0.021

Notes: proportion of the total effect that is mediated is 0.066; ratio of indirect to direct effect is 0.071; ratio of total to direct effect is 1.071. B refers the regression coefficient, SE refers the standard error, and Z refers the value of the Z test.

## Data Availability

The datasets used in the current study are available from the corresponding author on reasonable request.
